# The rate by which mortality increase with age is the same for those who experienced chronic disease as for the general population

**DOI:** 10.1093/ageing/afab085

**Published:** 2021-05-26

**Authors:** Marcus Ebeling, Roland Rau, Håkan Malmström, Anders Ahlbom, Karin Modig

**Affiliations:** Research Group Mathematical and Actuarial Demography, Max Planck Institute for Demographic Research, Rostock, Germany; Research Group Mathematical and Actuarial Demography, Max Planck Institute for Demographic Research, Rostock, Germany; Department of Sociology and Demography, University of Rostock, Rostock, Germany; C6 Institute of Environmental Medicine, Karolinska Institutet, Stockholm, Sweden; Swedish Orphan Biovitrum AB (Sobi), Stockholm, Sweden; C6 Institute of Environmental Medicine, Karolinska Institutet, Stockholm, Sweden; C6 Institute of Environmental Medicine, Karolinska Institutet, Stockholm, Sweden

**Keywords:** rate-of-ageing, chronic disease, cancer, diabetes, myocardial infarction, older people

## Abstract

**Background:**

Mortality doubles approximately every 6–7 years during adulthood. This exponential increase in death risk with chronological age is the population-level manifestation of ageing, and often referred to as the rate-of-ageing.

**Objective:**

We explore whether the onset of severe chronic disease alters the rate-of-ageing.

**Methods:**

Using Swedish register data covering the entire population of the birth cohorts 1927–30, we analyse whether being diagnosed with myocardial infarction, diabetes or cancer results in a deviation of the rate-of-ageing from those of the total population. We also quantify the long-term mortality effects of these diseases, using ages with equivalent mortality levels for those with disease and the total population.

**Results:**

None of the diseases revealed a sustained effect on the rate-of-ageing. After an initial switch upwards in the level of mortality, the rate-of-ageing returned to the same pace as for the total population. The time it takes for the rate to return depends on the disease. The long-term effects of diabetes and myocardial infarction amount to mortality levels that are equivalent to those aged 5–7 years older in the total population. For cancer, the level of mortality returns to that of the total population.

**Conclusion:**

Our results suggest an underlying process of ageing that causes mortality to increase at a set pace, with every year older we become. This process is not affected by disease history. The persistence of the rate-of-ageing motivates a critical discussion of what role disease prevention can play in altering the progression of ageing.

## Key Points

- The increase in mortality with age is a classical measure of ageing at the population level, often called the rate-of-ageing.
- Myocardial infarction (MI), diabetes and cancer increase the absolute level of mortality, but not the rate-of-ageing.
- Long-term mortality levels after MI or diabetes are similar to levels of those aged 5–7 years older in the total population.
- Cancer increases the level of mortality, but the long-term survivors return to the level of the total population with time.

## Introduction

Ageing corresponds to a progressive decline in physiological function with chronological age and the loss of reparability functions of cells, leading to an increase in death risk with age [1]. The increase in death risk, or more general, in mortality with age is a classic measure of ageing at the population level, often employed by biologists and demographers, and referred to as the rate-of-ageing [2, 3]. In fact, adult mortality has shown to double approximately every 6–7 years, which expresses that the death risk at adult ages increases exponentially by around 11% from one age to another [4]. The regularity of this rate across populations and over time is one of the major reasons why it has been hypothesised that the human rate-of-ageing is essentially the same across individuals [5]. This hypothesis integrates into a controversial debate about the opportunities to slow the human ageing process that can be traced back to antiquity [6–11].

A few studies have explored if the rate-of-ageing is altered by factors such as physical activity, sudden environmental changes or a combination of factors by investigating longevity vanguards [12–15]. These studies documented changes in the level of mortality, but not in the increase of mortality with age: the rate-of-ageing remained unaltered in these settings. Whether the rate-of-ageing can be altered in general has also been investigated in experimental settings using model organisms. In these studies, the organisms have been exposed to changes in diet or gene modifications [16, 17]. These interventions resulted in a considerable extension of lifespans, but changed the rate-of-ageing only marginally, if at all.

Following the same logic, but instead of studying how certain factors can slow down the rate-of-ageing, we wanted to explore how factors known to increase mortality affect the rate-of-ageing. Specifically, we suggest to test ‘if and how the occurrence of different diseases affects the rate-of-ageing?’ It is well known that severe disease like diabetes, myocardial infarction and cancer shorten human lives [18]. The shortening of lifespans operates through an increase in the level of mortality, but it is not known if these diseases also alter the speed with which mortality increases with age—the rate-of-ageing. For cancer, it has been shown that clinical ageing is observed earlier in cancer survivors than among healthy controls, or the general population [19, 20]. However, whether this is due to a shift towards a higher level on the scale for biological age or if the age trajectory has actually accelerated has not been shown.

Diseases can be linked to ageing in several ways. First, the mere onset of a disease is a stressor for the human body, which may cause the rate-of-ageing to go up. Second, disease can also be interpreted as a manifestation of underlying characteristics such as genetics or lifestyle, which affect individual health and as a consequence the rate-of-ageing. Third, the onset of a disease may indicate a threshold where the deterioration of the body has reached a critical—potentially life threatening—level, and as a consequence, ageing may be accelerated.

In this study, we investigate how mortality varies between populations that have experienced different chronic diseases. More specifically we explore how these diseases affect the level of mortality, as well as the relative increase with age, to see if the increase remains at around 11% per chronological year, or if it speeds up. The selected diseases, myocardial infarction, diabetes and cancer, represent different pathogeneses. Even though following upon an underlying artery disease, myocardial infarction can be considered as an acute event or ‘shock’. Diabetes develops over a longer time period without a clear onset, even if diagnosed at one point in time. It is associated with several adverse health consequences and an excess risk of mortality, even after adjusting for co-existing diseases [21, 22]. Finally, by looking at cancer, we include a disease that is not only the result of an accumulation of risk factors but also of pure chance by random mutations, even if the contribution of randomness has been debated [23, 24].

## Data and Method

### Data

Myocardial infarction was defined from hospital admissions with International Classification of Diseases (ICD) codes I21 and I22 in ICD-10 or 410 in ICD-9 from the Swedish National Patient Register. Information on cancer was collected from the Swedish Cancer Register [25–29]. This data covers all males and females over the age of 60 that lived in Sweden at any point in time between 1987 and 2017. We used the 1st occurrence of each diagnosis; and for myocardial infarction, we additionally conditioned on having survived the acute phase, which we defined as the first 28 days after diagnosis. For cancer, we used the date of diagnosis as the start of follow-up.

The subgroup of diabetics was retrieved from a cohort called Apolipoprotein-related Mortality Risk (AMORIS) cohort, which has been described in detail before [30]. All individuals in the AMORIS cohort were either healthy individuals referred for clinical laboratory testing as a routine part of yearly health check-ups through occupational health care or were outpatients referred for laboratory testing. The AMORIS cohort constituted a substantial part (about 35%) of the total population of Stockholm County at the time of the health examination and inclusion in the cohort. The follow-up of the AMORIS cohort allowed analysis until 2012. Diabetes was defined as meeting at least one of the three following criteria: (1) a diabetes diagnosis prior to baseline in the Swedish National Diabetes Register; (2) a fasting glucose value ≥7.0 mmol/l or an glycated hemoglobin A1c (HbA1c) ≥6.5% at the baseline examination; and (3) a hospital discharge diagnosis of diabetes prior to the baseline examination recorded in the National Patient Register.

Our analysis for all three diseases includes the cohorts born from 1927 to 1930. We pooled the four birth cohorts to increase population size. This allowed us to cover the ages 60–87 for myocardial infarction and cancer as well as the ages 60–82 for diabetes. In the case of cancer and myocardial infarction, we used the age at diagnosis as indicator for identifying the disease-specific population. For diabetes, diagnosis up to a specific age was used to identify the disease-specific population. This framework allowed us to count deaths and estimate person-years lived by years of age.

### Methods

Age-specific death rates for the three disease-specific subpopulations were estimated and compared with the death rates of the total population. Due to relatively small sample sizes, these observed death rates can exhibit large variability. Therefore, we estimated in addition a smooth mortality trajectory and corresponding standard errors [31]. The estimation of person-years lived during the age of onset could be heavily affected by imprecise measurements of the exact moment of disease onset. The smoothing approach has therefore been applied for the following age onward in the cases of cancer and myocardial infarction (e.g. smoothed estimates, for example, with age 60 as age at diagnosis rest on death counts and person-years lived for ages 61 and onward). This problem applies in principle also to diabetes. Due to the cumulative measurement of disease onset, the fraction at age 60 or 70 is negligibly small. Thus, we decided to analyse death rates from the respective ages onward.

We investigate the effects on the rate-of-ageing by assessing age-specific death rates. We plot those rates on the log-scale since this transforms the exponential increase at adult ages to a linear trajectory. The rate-of-ageing is the slope of this increase, and thus it expresses the relative increase of mortality with age. Sustained changes in the slope of the disease-specific mortality trajectories correspond to changes in the rate-of-ageing.

**Figure 1:**
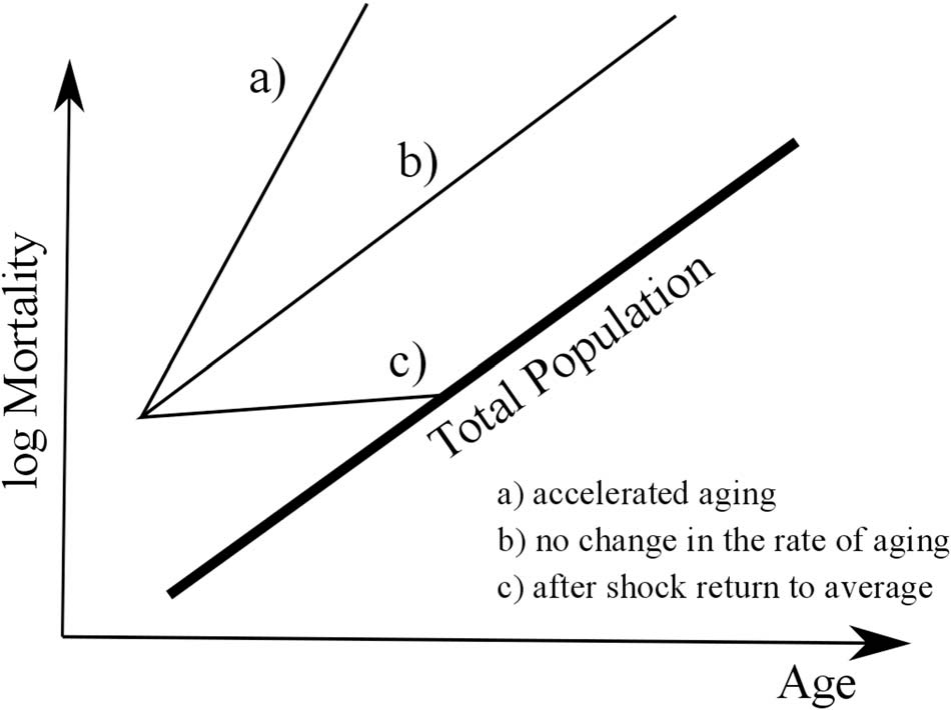
Potential effects of the disease onset on age-specific mortality.

Figure 1 illustrates the three possibilities of how the rate-of-ageing could be altered by a disease. Generally, we expect that mortality rises after the occurrence of the respective disease. Hence, mortality in all three scenarios exceeds those of the total population. In the 1st case (a), the occurrence of the disease results in an increase of the slope of mortality with age, and thus a change in the rate-of-ageing by accelerating ageing. In the 2nd case (b), the occurrence of the disease does not change the slope of mortality increase with age, and thus has no effect on the rate of ageing. In the 3rd case (c), the occurrence of the disease results in a sudden rise in mortality that is followed by a return to the average mortality level, and thus the disease would have no sustainable effect on the rate-of-ageing.

To illustrate the long-term mortality effects caused by the diseases, we use ages with equivalent mortality experience [32]. These ‘equivalent ages’ express mortality differentials in terms of the age where the specific mortality level is observed in the reference population. A hypothetical example: a diagnosis of disease *x* is associated with mortality levels, which are usually experienced 5 years later in the reference population. The equivalent ages are also a further tool to investigate the effect on the rate-of-ageing. According to the trajectories in Figure 1, the possible effect outlined under (a) would be accompanied by a consistently growing age gap, the possible effect outlined under (b) would be accompanied by a constant age gap and the possible effect outlined under (c) would be accompanied by a consistently declining age gap.

## Results

Table 1 describes the basic characteristics of the data used in this study. The analysis of diagnosis at age 60 rests on 320 myocardial infarction diagnoses (256 deaths), 1,246 cancer diagnoses (844 deaths) and 610 diabetes diagnosis (348 deaths) for females. The corresponding numbers for males are 1,083 myocardial infarction diagnoses (905 deaths), 1,189 cancer diagnoses (847 deaths) and 1,274 diabetes diagnosis (844 deaths). The analysis based on diagnoses at (or before) age 70 rests on 645 myocardial infarction diagnoses (452 deaths), 1,703 cancer diagnoses (1,046 deaths) and 1,561 diabetes diagnosis (600 deaths) for females, as well as 1,218 myocardial infarction diagnoses (882 deaths), 2,418 cancer diagnoses (1,669 deaths) and 2,700 diabetes diagnosis (1,222 deaths) for males.

**Table 1:** Number of cases, deaths and person-years at risk by disease and age at diagnosis, males and females, birth cohorts 1927–30

Disease	Sex	Age at diagnosis	Age range follow-up	Number of cases	Person-years at risk	Deaths
Myocardial infarction	F	60	61–87	320	4,724.681	256
	M	60	61–87	1,083	15,156.855	905
	F	70	71–87	645	6,737.066	452
	M	70	71–87	1,218	11,689.711	882
Cancer	F	60	61–87	1,246	15,152.592	844
	M	60	61–87	1,189	9,332.779	847
	F	70	71–87	1,703	12,687.3	1,046
	M	70	71–87	2,418	15,701.72	1,669
Total population	F	–	60–87	–	3,293,036	98,799
	M	−	60–87	−	3,985,292	120,734
Diabetes	F	<61	60–82	610	10,618.16	348
	M	<61	60–82	1,274	20,057.12	844
	F	<71	70–82	1,561	14,224.97	600
	M	<71	70–82	2,700	20,792.4	1,222
Total population AMORIS	F	–	60–82	–	380,001.5	6,070
	M	–	60–82	–	369,065	9,498

Notes: Myocardial infarction and cancer are identified in nationwide population registers, and diabetes cases are extracted from the AMORIS cohort that includes individuals primarily from the Stockholm County, Sweden. The total population, which provides reference mortality, are thus different for diabetes compared with the other two diseases.

**Figure 2:**
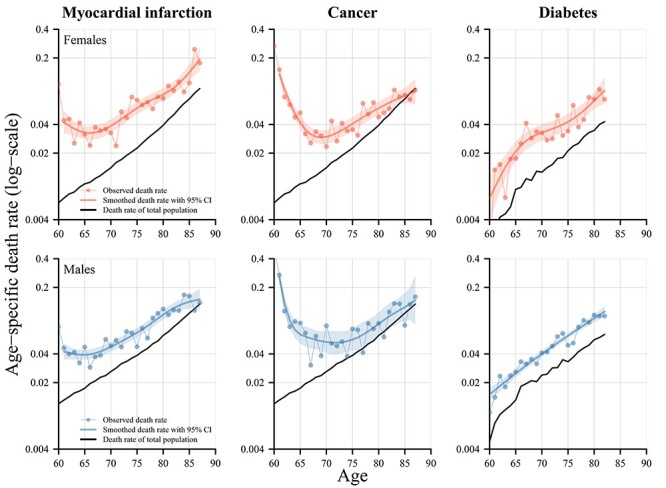
Age-specific death rates for subpopulations with a diagnosis of either myocardial infarction, cancer or diabetes at (and before) age 60 and the respective total population, females and males, birth cohorts 1927–30, Sweden. Notes: The estimates of myocardial infarction and cancer are based on nationwide population registers, whereas the estimates for diabetes are based on the AMORIS cohort that includes only individuals from Stockholm County, Sweden. The total population are thus different for diabetes compared with the other two diseases. For myocardial infarction, the start of follow-up is onset at age 60 plus 28 days. For cancer, follow-up starts after detection/diagnosis of any cancer diagnosis at age 60, and in the case of diabetes, follow-up is age 60 for individuals with a diabetes diagnosis at age 60 or any earlier age.

Figure 2 shows the age-specific death rates from age 60 and onwards of the subpopulations for the three respective diseases and the total population.

The mortality curves suggest that none of the diseases resulted in a sustained change in the slope of mortality increase with age, and thus we cannot detect any changes in the rate-of-ageing. Myocardial infarction at age 60 results in an initial rise in the level of mortality and higher levels of mortality in the long run, but the mortality increase with age occurs almost in parallel to that of the total population. Individuals with a cancer diagnosis at age 60 experience a similar sharp upturn in the level of mortality in the years after the diagnosis. In the long run, the pattern, however, differs from those of myocardial infarction. The death rates 5–10 years after the diagnosis of cancer reveal a continuous convergence towards mortality of the total population. During this phase the rate-of-ageing is slower than that of the total population, which likely results from the selection process towards individuals that survive their cancer. In the case of a diabetes diagnosis at age 60 or earlier, the subpopulation experiences higher levels of mortality, but a parallel increase with age.

**Figure 3:**
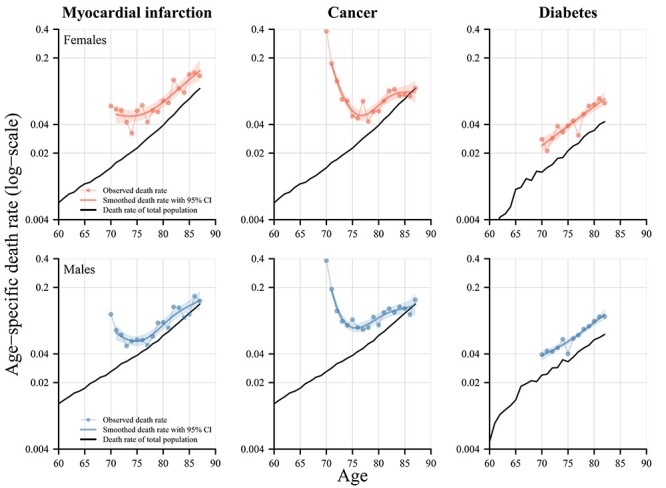
Age-specific death rates for subpopulations with a diagnosis of either myocardial infarction, cancer or diabetes at (and before) age 70 and the respective total population, females and males, birth cohorts 1927–30, Sweden. Notes: The estimates of myocardial infarction and cancer are based on nationwide population registers, whereas the estimates for diabetes are based on the AMORIS cohort that includes only individuals from Stockholm County, Sweden. The total population are thus different for diabetes compared with the other two diseases. For myocardial infarction, the start of follow-up is onset at age 70 plus 28 days. For cancer, follow-up starts after detection/diagnosis of any cancer diagnosis at age 70, and in the case of diabetes, follow-up is age 60 for individuals with a diabetes diagnosis at age 70 or any earlier age.

The general patterns observed for diagnosis at (or before) age 60 hold also if a diagnosis at (or before) a later age is considered. Figure 3 shows the results at (or before) age 70. The reference populations are the same as in Figure 2. The general patterns of age-specific mortality for all three diseases shown in Figure 2 are very similar to those observed in Figure 3. The diagnosis of myocardial infarction and cancer at age 70, however, resulted in lower excess mortality compared with levels observed for a diagnosis at age 60. For diabetes, the level of excess mortality remains nearly unchanged compared with the level of diagnosis at or before age 60.

**Figure 4:**
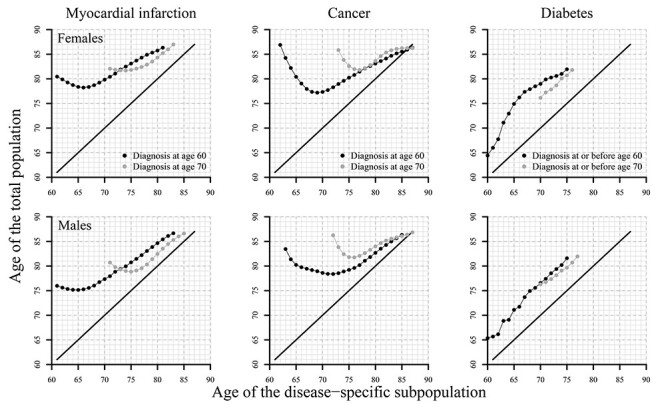
Ages of similar mortality levels for subpopulations with a diagnosis of either myocardial infarction, cancer or diabetes at (and before) age 60 and 70 compared with mortality of respective total population, females and males, birth cohorts 1927–30, Sweden. Notes: The estimates of myocardial infarction and cancer are based on nationwide population registers, whereas the estimates for diabetes are based on the AMORIS cohort that includes only individuals from Stockholm County, Sweden. The total population, which provides reference mortality, are thus different for diabetes compared with the other two diseases. Note that if a specific death rate is outside the range of observed death rates for the total population, no equivalent age can be calculated, but the equivalent age can be quantified as being higher than the highest age observed for the total population (e.g. equivalent age is higher than 87).

In Figure 4, death rates of the disease-specific populations are translated into differences in years of age, so called equivalent ages. If the curves were on the black solid line across the diagonal, both the disease specific and the total population would experience the same mortality levels at each age. Points above this line express that death rates observed at a specific age for the disease-specific population are always observed at a later age in the total population.

All estimates in Figure 4 are above the ‘equivalent-age-line’. The age-specific death rates of individuals with a given disease thus are always observed at younger ages than in the total population. The magnitudes of age differences differ substantially across age and also between the disease-specific populations.

After the immediate impact is diminished, a myocardial infarction at age 70 results in a persistent age differences of 2.5 years for males and 4 years for females. Death rates observed at age 80 for males and females with a myocardial infarction diagnosis at age 70 thus correspond to death rates observed at age 82.5 for males and age 84 for females.

After the immediate effect of a cancer diagnosis, the observed age differences show a continuous decline. For instance, the age gap of 4 years at age 75 for males with a cancer diagnosis at age 60 declines to around 1 year for death rates observed at age 85 for this disease-specific population. The pattern of females is similar to those of males.

Diabetes shows generally a nearly constant age difference of about 5–6 years in both analysis settings and males and females. Females with a diagnosis before or at age 60 may be an exception to this finding because they exhibit a concave pattern. However, this is likely driven by some peculiarities in the data, and thus should be interpreted with caution. Despite this exception, for instance, death rates that are observed at age 70 for diabetics correspond to death rates the total AMORIS cohort experiences at ages 76.

## Discussion

We found that none of the analysed diseases had a sustained effect on the rate-of-ageing. Given the scheme presented in Figure 1, the occurrence of myocardial infarction or diabetes resulted in a shape that is best described by the dynamics under (b), a rise in the level of mortality but no change in the rate-of-ageing, whereas the occurrence of cancer revealed a pattern best described under (c), after a short increase, a return to the mortality level of the total population. The general pattern holds true for different ages of diagnosis and, in the case of cancer, also for specific cancer types (results are not shown but available upon request from the corresponding author). Both diabetes and myocardial infarction result in persistently elevated death risks that are equivalent to those aged 5–7 years older in the total population. The age gap is lower if the myocardial infarction occurs later in life. This stability in the age gaps illustrates again the negligible effect of the diagnosis of these two diseases on the rate of ageing. Cancer, on the other hand, allows a convergence of the elevated risk, even if it takes many years. Moreover, particularly for myocardial infarction, females exhibited a stronger effect on mortality compared with males. This sex difference illustrates that a myocardial infarction at age 60 and even at age 70 may be considered early for females, and thus identifies a particularly vulnerable group of individuals, whereas this might not be the case for males.

Our results resemble past findings about the lack of plasticity of the human rate-of-ageing on the population level. Previous studies focused on the effect of different extrinsic factors, such as environmental changes or dietary restrictions, and intrinsic factors, such as gene manipulation, on the population-level manifestation of ageing [12–17]. Although our approach does not allow any conclusions about the underlying mechanism, we used an indicator (disease) that combines extrinsic factors, such as lifestyle, and intrinsic factors, such as genetic disposition. Extrinsic and intrinsic factors combined result in the vulnerability and ultimately in the development of a disease. Moreover, the development of the respective diseases can also be interpreted as different types of manifestations of the physiological decline with age, and thus as different pathways of human ageing.

Human ageing can be measured in various ways, and it is controversially debated how it should be measured. It has been argued, for instance, that mortality could be considered to be the most robust indicator of health, and thus of the progression of senescence [5]. However, scholars seek to find more sensitive indicators to measure ageing [33–36]. With the relative increase of mortality with age, we employed an indicator that is solely based on the mortality experience of a population. Thus, our results contribute to better understand the progression and plasticity of the ageing process, but in view of the complexity of the ageing process, it cannot cover all aspects.

Although the analysis revealed no changes in the rate-of-ageing, the occurrence of the diseases altered mortality in a unique way. The patterns of myocardial infarction and diabetes suggest that both diseases have negative irreversible effects on health and ageing, whereas the pattern of cancer suggests that negative long-term effects can eventually be reduced to a minimum, given that individuals survive the severe phase. How the effect of age on mortality changes with the diagnosis of any of the included diseases, and how this translates into equivalent ages, emphasise the difference between chronological and biological age. It may be of clinical relevance to note that the diagnosis of diabetes and myocardial infarction results, on a population level, in an increase of the mortality risk at any given age by an approximate constant factor. The corresponding levels are equivalent to the mortality risks observed 5 years later in the general population. For myocardial infarction, the factor, however, declines with increasing age at diagnosis. This is not due to a faster ageing process by chronological age, but because these groups are pushed upward to a higher level of mortality.

## Conclusion

Our results support the concept of an underlying process of ageing causing mortality to increase with every year older we become and suggest that this process is independent of disease history. That is, some groups are more vulnerable than others and therefore experience a higher level of mortality, but the effect of age hits everyone in the same manner, it is just a matter of time.
